# Omics Technologies to Enhance Plant Based Functional Foods: An Overview

**DOI:** 10.3389/fgene.2021.742095

**Published:** 2021-11-08

**Authors:** Spurthi N. Nayak, B. Aravind, Sachin S. Malavalli, B. S. Sukanth, R. Poornima, Pushpa Bharati, Kathleen Hefferon, Chittaranjan Kole, Naveen Puppala

**Affiliations:** ^1^ Department of Biotechnology, University of Agricultural Sciences, Dharwad, India; ^2^ Department of Food Science and Nutrition, University of Agricultural Sciences, Dharwad, India; ^3^ Department of Microbiology, Cornell University, Ithaca, NY, United States; ^4^ President, International Phytomedomics and Nutriomics Consortium (ipnc.info), Daejeon, South Korea; ^5^ New Mexico State University-Agricultural Science Center at Clovis, New Mexico, NM, United States

**Keywords:** nutrition, genomics, transgene, functional foods, nutraceuticals

## Abstract

Functional foods are natural products of plants that have health benefits beyond necessary nutrition. Functional foods are abundant in fruits, vegetables, spices, beverages and some are found in cereals, millets, pulses and oilseeds. Efforts to identify functional foods in our diet and their beneficial aspects are limited to few crops. Advances in sequencing and availability of different omics technologies have given opportunity to utilize these tools to enhance the functional components of the foods, thus ensuring the nutritional security. Integrated omics approaches including genomics, transcriptomics, proteomics, metabolomics coupled with artificial intelligence and machine learning approaches can be used to improve the crops. This review provides insights into omics studies that are carried out to find the active components and crop improvement by enhancing the functional compounds in different plants including cereals, millets, pulses, oilseeds, fruits, vegetables, spices, beverages and medicinal plants. There is a need to characterize functional foods that are being used in traditional medicines, as well as utilization of this knowledge to improve the staple foods in order to tackle malnutrition and hunger more effectively.

## Introduction

**FIGURE 1:**
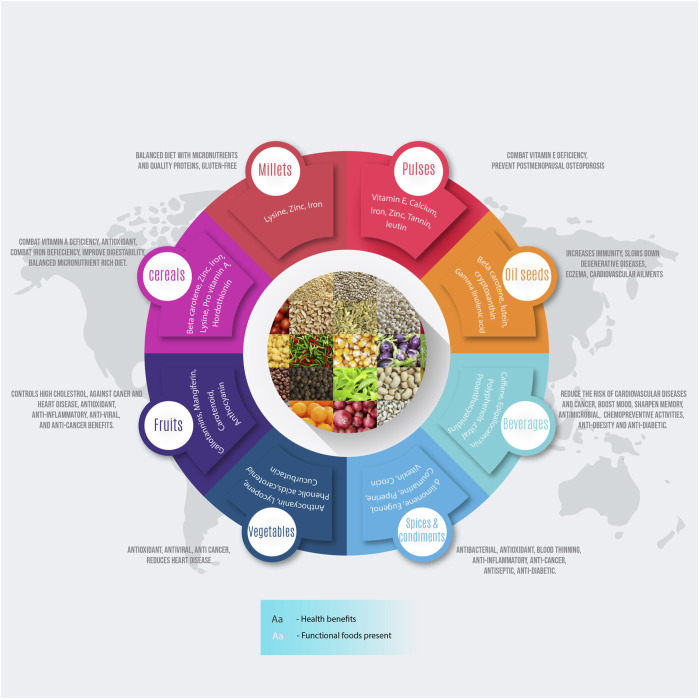
An infographics showing the functional components of the food and their potential health benefits to human beings.

To address global food and nutritional security, there is a need to increase the agricultural production and nutritive value of food. Assured access to nutritionally adequate and safe food is essential for attaining the nutritional security. With urbanization and changing food habits, “smart foods with higher nutrition per bite” is the need. The awareness of utilization of these foods for prevention and treatment of certain diseases prompted the researchers to discover active compounds that render health benefits. Foods that have an additional physiological benefits besides providing basic nutritional needs were first referred to as “functional foods” in Japan in the mid-1980s. Broadly, functional foods can be categorized according to the active components that have health benefits. Based on their origin, they can be classified as naturally derived products (plant or animal sources) or synthetic products (synbiotics, nutraceuticals) (Mohanty and Singhal, 2018). The functional products from plant origin include phytochemicals such as polyphenolic compounds, alkaloids, flavonoids, carotenoids, saponins, allyl sulfides, catechins, nutraceuticals, etc. (Table 1; Figure 1). There are clear evidences from epidemiological studies and clinical trials that a plant-based diet can reduce the risk of chronic diseases and disorders such as cancer (Velmurugan et al., 2005; Aghajanpour et al., 2017; Sayeed et al., 2017), diabetes (Hannan et al., 2007; Ballali and Lanciai, 2012; Alkhatib et al., 2017), obesity (Hill and Peters, 2002; Riccardi et al., 2005; Baboota et al., 2013), cardiovascular ailments (Alissa and Ferns, 2012; Hamid and Abd Hamid, 2019) and other effects on human health (Lobo et al., 2010). Most of the functional foods with scientific supporting evidence are the native/familiar foods that were used in traditional medicine for generations (Hasler, 1998; Fokunang et al., 2011; Abbott, 2014).

**TABLE 1:** Functional compounds and their health benefits.

Sl. No	Compound	Health benefits	References
1	Tocopherols, β-carotene	Antioxidants, reduce the risk of heart diseases and few types of cancers and protect from age-related muscular degeneration	Sies and Stahl, 1995; Gul et al., 2015, Jacobo- Valenzuela et al., 2011
2	α-linolenic acid	Cardioprotective in nature, modulation of an inflammatory response, and improves central nervous system functions	Stark et al. (2008)
3	Astaxanthin	Antioxidant and anti-inflammatory improves blood circulation and brain functions, promote an integrated immune response	Kidd, (2011)
4	Anthocyanins	Acts as dietary antioxidants helps to prevent neural diseases, cardiovascular problems, diabetes, inflammation and many other diseases	Yousuf et al. (2016)
5	Tannins	Antioxidant, anti-inflammatory, anticancerous, antiallergic, antihelminthic and antimicrobial activities	Sharma et al. (2019)
6	β-glucan	Beneficial role in insulin resistance, dyslipidemia, hypertension, and obesity	El Khoury et al. (2012)
7	Lycopene	Antioxidant, anticancer, protect against cardiovascular diseases, modulation of inflammatory responses, cholesterol reduction	Thies et al. (2017)
8	Flavonoids	Antioxidant, prevention of coronary heart diseases, hepatoprotective and anti-cancer activity	Yao et al., 2004; Yadav et al., 2020, Basu et al., 2018
9	Vitamin C	Prevent scurvy, coronary heart diseases stroke and cancer	Granger and Eck (2018)
10	Alkaloids	Analgesic, antipyretic, antioxidants, anti-inflammatory, improves brain functioning, antidiabetic and helps to treat gastroenteritis and chronic diseases	Derosa et al., 2016; Street et al., 2017, Adams et al., 2014
11	Saponins	Lowers blood lipids, lower blood glucose response and cholesterol levels, reduce cancer risks	Shi et al. (2004)
12	Eugenol	Antioxidants, anti-inflammation, helps to control hyperglycemia, elevated cholesterol levels, neural disorders and cancer. Also, possess antimicrobial agent	Khalil et al. (2017)
13	Polyphenols	Neuroprotectve, anti-aging, Antioxidant, anti-inflammatory	Lau et al. (2005)
14	Isothiocyanates	Lowers the risk of liver, breast, lung cancers	Aghajanpour et al., 2017; Mohanty and Singhal, 2018; Kartikey et al., 2019
15	Phytosterols	Anticancer, antibacterial, antiviral, and cholesterol-lowering activity	Chongtham et al. (2011)

The scientific advances and next generation sequencing technologies available in recent years have impacted significantly on crop breeding and food science (Varshney et al., 2009; Kato et al., 2011). There is a lot of scope to utilize these technologies to understand the functional compounds that have health benefits and to improve the crops with respect to its nutritional status along with productivity related traits. Utilization of different omics technologies in research related to food and nutrition with the objective of improving the human health and well-being is referred as foodomics (Capozzi and Bordoni, 2013). An integrated use of omics technologies approaches to increase the nutrient potential of any crop, further applications in food processing and formulations can influence the nutritional security to greater extent (Bagchi et al., 2015; Tian et al., 2016).

**FIGURE 2:**
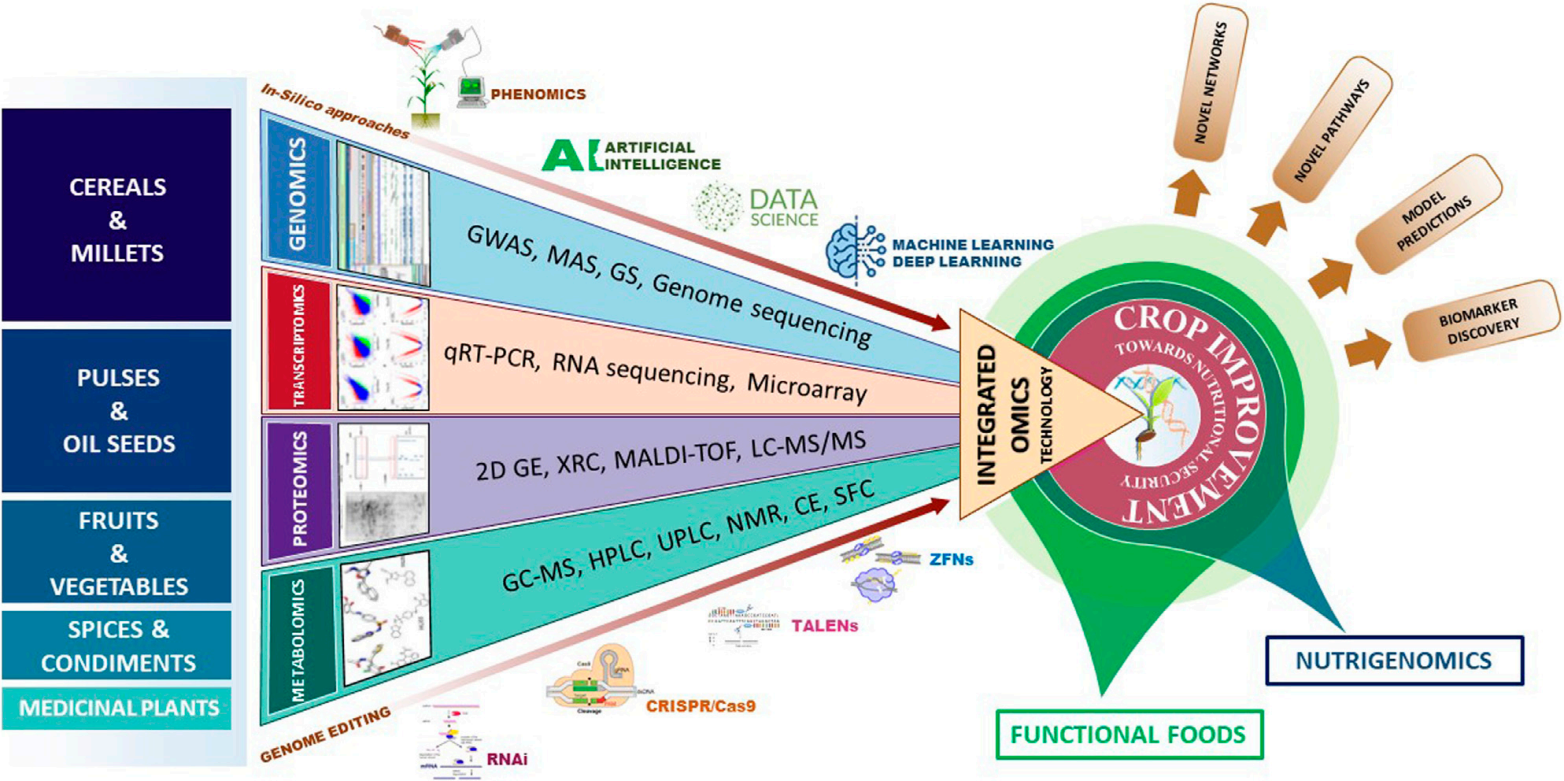
Integrated omics approaches for enhancing functional foods. In this figure, the crop categories specified are orderly arranged in descending manner with respect to the utilization of omics technologies to improve functional foods. Abbreviations: GWAS, Genome-Wide Association Studies; MAS, Molecular Assisted Selection; GS, Genomic Selection; qRT-PCR, Quantitative Real Time polymerase Chain Reaction; 2D GE, 2-Dimensional Gel electrophoresis; XRC, X-Ray Crystallography; MALDI-TOF, Matrix-Assisted Laser Desorption/Ionization-Time Of Flight; LC-MS/MS, Liquid Chromatography—Mass Spectrophotometry; GC-MS, Gas Chromatography—Mass Spectrophotometry, HPLC: High Performance Liquid Chromatography; UPLC, Ultra Performance Liquid Chromatography; NMR, Nuclear Magnetic Resonance; CE, Capillary Electrophoresis and SFC, Supercritical Fluid Chromatography; RNAi, RNA interference; TALENs, Transcription Activator-Like Effector Nucleases; ZFNs, Zinc Finger Nucleases.

The omics discipline comprises of four major broad areas like genomics, transcriptomics, proteomics and metabolomics. Integrated use of the omics technologies provides a holistic approach to study the systems biology (Pazhamala et al., 2021). Genomics includes the sequencing of whole genomes, assembly and annotation of the sequences, study of the genes, identification and development of molecular markers and quantitative trait loci (QTLs) for target traits, genomics assisted breeding, genomic selection, etc. (Varshney et al., 2005; Kole et al., 2015; McGuire et al., 2020). Transcriptomics deals with the dynamic expression of gene products in specific tissue at particular stage. The study of differential expression is quantified by using different molecular biology tools such as RNA sequencing, microarrays, Serial analysis of Gene Expression (SAGE), qRT-PCR, etc. While microarray, SAGE and qRT-PCR technologies determine the abundance of defined transcripts, the RNA-sequencing utilizes the advantage of high-throughput sequencing to identify the novel transcripts (Lowe et al., 2017). Proteomics can be effectively used to study protein structure, function, and interaction with other proteins or ligands such as bioactive compounds. Advanced techniques like Matrix-assisted laser desorption/ionization Time of flight (MALDI-TOF) and Liquid chromatography coupled to mass spectrometry (LC-MS) are able to detect expression of specific proteins. Metabolomics identifies and quantifies specific metabolites present in a sample. Metabolomics can be beneficial for quantification of biologically active compounds, food fingerprinting, and food profiling. Techniques like Gas chromatography coupled to mass spectrometry (GC-MS), Liquid chromatography coupled to mass spectrometry (LC-MS), Inductive couple plasma (ICP), nuclear magnetic resonance (NMR), Near infrared spectrometry (NIR) have been used for characterization of metabolites (Prakash et al., 2018; Kumar et al., 2019). Besides these omics approaches, genome editing tools like RNAi, CRISPR/Cas9, TALENs, ZFNs can be utilized to improve the crop plants. Use of computational and bioinformatics tools is indispensable while using all the above mentioned technologies. Advances in data science with applications of artificial intelligence and machine learning has enabled deep learning of the data for better understanding of the biological processes and crop prediction modelling in genomic selections (Figure 2). In this review, we discuss about the utilization of omics technologies in determining and enhancing the active food compounds in major crop plants including cereals, millets, pulses, oil seeds, fruits, vegetables, spices and medicinal plants.

### Cereals

Cereals are the major part of our daily diet and source of carbohydrates but lack an adequate amount of nutrition in terms of vitamins, and essential amino acids (Munck, 1972). Hence there is a need to improve the quality and nutritional parameters of cereals. Recent advances in genomics and genetic engineering are useful in targeted improvements especially by improving the quality and nutritional value in crop plants (Sedeek et al., 2019). Several omics technologies have been used to improve rice, wheat, barley especially for disease resistance and improving the yield of the crops (Zenda et al., 2021). However, there are only a few reports related to deciphering the functional compounds in cereal crops using modern biotechnological tools (Table 2). For instance, rice is improved with higher carotenoid content leading to increased Vitamin A (Dubock, 2019) and biofortified with micronutrients like Fe and Zn (Welch and Graham, 2004; Trijatmiko et al., 2016). A genetic engineering approach was successfully used to develop “Golden Rice” with significant levels of β-carotene that will help to combat vitamin A deficiency. Ye et al. (2000) and Shao et al. (2011) reported the marker loci/QTLs underlying the naturally occurring variations of grain color and nutritional quality traits in 416 rice germplasm accessions, including 361 white rice, 50 red rice, and six black rice across 41 marker loci. These markers could be further used for marker-assisted breeding to improve rice for nutritional qualities. The efforts were also made to dissect the nutrient traits especially Fe, Zn and anthocyanin content using genome-wide association studies on diversity panel consisting of 156 accessions of colored rice (Descalsota-Empleo et al., 2019). QTLs for functional components like phenolic content, flavonoid content and antioxidant capacity were identified using 127 double haploid lines developed through anther culture (Jin et al., 2009). Genome editing tools like CRISPR-Cas9 have also been utilized to enhance the amylose content (Sun Y. et al., 2017b). There are also efforts to develop fragrant rice by knocking out of betaine aldehyde dehydrogenase (*BADH2*) gene using TALEN technology (Shan et al., 2015).

**TABLE 2:** Study of functional foods in cereals and millets using biotechnological approaches.

S. No	Crop	Functional food	Gene(s)/QTL(s)	Methodology	References
1	Rice	β-carotene	Daffodil &*crtI* gene	Transgenic and expression studies	Beyer et al. (2002)
			G*t*HMG1, G*Zm*Psy1 and G*Pa*CrtI1 genes		Tian et al. (2019)
		Fe and Zn	Ferritin	Transgenic and interval mapping	Lucca et al., 2002; Vasconcelos et al., 2003; Zhang et al., 2014
			genes (*Os*MTP6, *Os*NAS3, *Os*MT2D, *Os*VIT1, and *Os*NRAMP7) and 7 QTLs for each Fe and Zn	GWAS	Descalsota et al. (2018)
			48 MQTLs and 8 genes related to grain Fe and Zn concentration	MQTL analysis	Raza et al. (2019)
		α-linolenic acid rich	chimeric gene consisting of a maize *Ubi1-P-int* and a soybean *GmFAD3* cDNA	Transgenics	Anai et al. (2003)
		Astaxanthin	*s*ZmPSY1, *s*PaCrtI, *s*CrBKT, and *s*HpBHY genes	Transgenics	Zhu et al. (2018)
		α-tocopherol	*Os*GGR2 gene	RNA interference	Kimura et al. (2018)
		Phytic acid	OsITP5/6K-1 gene	RNA interference	Karmakar et al. (2020)
		Resistant starch	*sbe3-rs* gene	MAS	Yang et al. (2020)
2	Wheat	Micronutrients and Vitamins	*Gpc-B1* gene and DArT markers	MAS	Distelfield et al., 2006; Uauy et al., 2006
		Zn, Fe, Cu, Mn, Se rich	QTLs for Zn, Fe, Cu, Mn, Se	Interval mapping	Pu et al. (2014)
		Anthocyanins	*Ba* gene *Pp3 and Pp-D1* genes	MAS	Gordeeva et al., 2019; Gordeeva et al., 2020
3	Wheat and Barley	PUFAs	Artificial D6-desaturase gene	Transgenics using the biolistic method	Čertík et al. (2013)
4	Sorghum	Lysine	*BHL-9*	Transgenics	Zhao et al. (2003)
		Protein	*hl* gene and P721 opaque gene	Mutation breeding and MAS	Axtell et al., 1979; Welch and Graham, 2004
		Vitamin A	Prolamin and lysine alpha-ketoglutarate reductase genes z	Transgenics	Lipkie et al. (2013)
		Fe and Zn	QTLs and candidate genes like *CYP71B34, ZFP 8*	QTL mapping	Kotla et al. (2019)
		starch and amylose	Grain quality/starch pathway genes *Sh2, Bt2, SssI, Ae1, and Wx*	GWAS	De Alencar Figueiredo et al. (2010)
		Tannin antioxidant	*tan-1, tan-1a* and *tan-1b*	GWAS and expression analysis	Wu et al. (2012)
		β-carotene andZeaxanthin	3 QTLs for β carotene and 4 QTLs forZeaxanthin	GWAS	Cruet-Burgos et al. (2020)
5	Maize	Vitamin C and E rich	DHAR cDNA	Transgenics	Chen et al. (2003)
		pro-vitamin A	*crtB* and *crtI*	Transgenics	Aluru et al. (2008)
			lcyE	MAS	Yang et al. (2018)
			*lcy*E, crtRB1, and o2	MABB	Sagare et al. (2019)
		Fe and Zn	SNPs associated with kernel Fe and Zn content	GWAS and QTL mapping	Hindu et al. (2018)
6	Barley	Hordothionin rich	Hordothionin	Mutation breeding and MAS	Rao et al. (1994)
		β-glucan	*Csl* genes and QTLs	GWAS and paired-end-RNA sequencing-based transcriptome	Cai et al., 2013; Chen et al., 2014; Shu and Rasmussen 2014
		Malting protein	13–30 candidate genes like *metallothionein, α-amylase, α-glucosidase, limit dextrinase, and β-ketoacyl synthase*	cDNA array-based gene expression analysis and SAGE	White et al., 2006; Lapitan et al., 2009
		Palatable and easily digestible	starch branching enzymes SBEIIa and SBEIIb	RNAi technology	Regina et al. (2010)
		—	20 QTLs associated with TPC, FLC and AOA	GWAS	Han et al. (2018)
7	Oats	β-glucan	QTL’s for β-glucan	GWAS, MAS, QTL mapping	Gazal et al. (2014)
8	Pearl millet	Fe and Zn	QTLs (11 for Fe and 8 QTLs for Zn)	QTL mapping	Kumar et al. (2018)
9	Finger millet	Calcium	Calmodulin and *Cax1* transporter genes	Differential expression/accumulation	Kumar et al. (2014)
10	Foxtail millet (*Setaria italica*)	storage associated genes	storage associated genes and noncoding RNAs	Transcriptome analysis	Qi et al. (2013)

Similar to rice, there are many reports to utilize the omics approaches in wheat to improve the nutrition and functional components. To list a few, an enriched wheat with high vitamin A content was developed by transforming the two bacterial carotenoid biosynthetic genes *CrtB* and *CrtI* into wheat cultivar Bobwhite (Wang et al., 2014). In addition to this, candidate genes involved in carotenoid biosynthesis and catabolism have been elucidated using GWAS studies in wheat (Colasuonno et al., 2017). Genomic regions for the color, carotenoids, and polyphenol oxidase activity of flour in wheat have been studied using linkage-based QTL analysis (Zhao et al., 2013). Hussain et al. (2017) reported QTLs for several nutrients, including Zn, Fe, Mn, Cu, Ca, Mg, etc. under saline conditions. In wheat, candidate genes for enhancing the grain Zn content have been identified by GWAS using high-density genotyping arrays on 369 wheat genotypes (Alomari et al., 2018). Genetic improvement in grain quality and micronutrients has been instrumental in quality breeding for wheat (Distelfeld et al., 2006; Balyan et al., 2013; Pu et al., 2014). Apart from this, there is also a need to utilize genomics approaches to decrease heavy metal (for example, Cadmium) uptake (Knox et al., 2009) and improve digestibility with reduced flatulence (Sharma et al., 2002). Genetically modified maize and wheat have showed increased accumulation of folate (Vitamin B9) levels (Liang et al., 2019).

Many economically backward countries rely on crops such as sorghum and maize as their staple food. In maize, biofortification with micronutrients like Zn and Fe (Zhao, 2007), enhanced β-carotene (Muthusamy et al., 2014), and amino acids like Lysine (Mertz et al., 1964; Shetti et al., 2020) have been carried out to ensure the nutritional security. To dissect the genomic regions for various metabolites in maize (*Zea mays*), a metabolome-based GWAS was carried out (Zhou et al., 2019). An integrated omics-based mapping to unravel flavonoid biosynthesis was also attempted in maize (Jin et al., 2017). There have been extensive efforts to breed for quality protein maize (QPM) with nearly as twice lysine and tryptophan content than the usual (Gibbon and Larkins, 2005). In addition to this, a transgenic approach has been used to increase protein by reducing zein content (Huang et al., 2006), and increasing Provitamin A content (Aluru et al., 2008). CRISPR-Cas9 and TALEN approaches have been used in maize to reduce phytic acid content, a food inhibitor that chelates micronutrients and prevents their bioavailability for mono gastric animals, including humans (Liang et al., 2014).

The reports related to the use of omics approaches to enhance functional compounds in barley (*Hordeum vulgare*) and oats (*Avena sativa*) are limited as compared to major cereals. In barley, the β-glucan content greatly improves the malting properties and its presence has been found to increase palatability (Chen et al., 2014). A gene expression study using SAGE analysis identified six proteins associated with the malting property (White et al., 2006). Other functional compounds like total polyphenols, flavonoids, and antioxidant properties were studied in 67 cultivated and 156 Tibetan wild barley accessions using GWAS (Han et al., 2018). In oats, a GWAS study was conducted in a global germplasm collection to identify molecular markers associated with β-glucan content (Newell et al., 2012; Gazal et al., 2014).

### Sorghum and Millets

Sorghum and millets are small-grained cereals and seed grasses that are traditional staple foods in African and Asian countries. In recent times, sorghum and millets are being utilized as an alternative to major cereals because of their higher nutritional, mineral, dietary fiber content along with climate-resilient nature. Besides, they are gluten-free and play a pivotal role in preventing and curing several lifestyle health issues like diabetes (Anitha et al., 2021). The genetic and genomic resources have been developed in some of the small millets (Vetriventhan et al., 2020) and efforts to utilize the genomic tools to improve the nutrient components are underway (Table 2). The nutraceutical property is mainly based on the kernel color in these crops. In this regard, QTL analysis of endosperm color and carotenoid (provitamin A) content in sorghum grains utilized in breeding high provitamin sorghum crop (Fernandez et al., 2008). Another effort using GWAS analysis with 404,628 SNP markers identified novel marker-trait association for polyphenols in a global diversity panel of 381 sorghum accessions (Rhodes et al., 2014).

There are limited efforts to utilize genomics tools in all other millets for improving the nutritional properties. The biofortification of millets seemed to be a good option for improving the nutritionally rich millets (Vinoth and Ravindhran, 2017). The QTLs controlling the content of micronutrients like Zn and Fe were identified in pearl millet (Kumar et al., 2018; Govindraj et al., 2019). Finger millet (*Eleusine coracana*) has been studied at various stages of growth and development using transcriptomics and was found to have high absorption and accumulation of calcium during grain development (Mirza et al., 2014). Glucosinolates in millets were found to reduce carcinogen-DNA interaction resulting in detoxification. Similarly, the isoflavones (phytoestrogens), genistein, and daidzein were found to reduce the incidence of many cancers, coronary heart diseases, and osteoporosis (Bandyopadhyay et al., 2017)**.** The bioavailability of the micronutrients present in millets needs to be elucidated and utilized in crop improvement.

### Pulses and Oilseeds

Pulses are a rich source of protein, with low fat, high fiber content and low glycemic index. Soluble fiber helps to decrease blood cholesterol levels and control blood sugar levels, and insoluble fiber helps with digestion. The biotechnological application for nutritional improvement mainly concentrates on enriching micronutrients and vitamins in pulses (Table 3). Pulses are known for the functional component saponins and several health benefits associated with them (Singh et al., 2017). Although pulses have been studied for several biotic and abiotic stresses at the molecular level, there are very few reports related to the genetic dissection of antioxidant activity and nutrition-related traits. Biofortification of pulses with Fe and Zn in lentils, chickpeas and field pea (*Pisum sativum*) has been carried out to address global malnutrition and micronutrient deficiencies (Thavarajah and Gupta, 2014). In chickpea, the GWAS study conducted in 94 diverse chickpea genotypes showed eight SNPs associated with Fe and Zn content in the seeds (Diapari et al., 2014). Similarly, a GWAS study in lentils identified two tightly linked SNP markers for Fe and Zn content (Khazaei et al., 2017).

**TABLE 3:** Study of functional foods in pulses and oilseeds using biotechnological approaches.

S. No	Crop	Functional food	Gene(s)/QTL(s)	Methodology	References
1	Soybean	Vitamin E	21 QTLs	QTL mapping	Li et al. (2010)
		α-tocopherol	6 QTLs associated with α-tocopherol content	QTL mapping	Park et al. (2019)
		—	19 QTLs were identified	GWAS	Sui et al. (2020)
		Tocopherol and tocotrienol	At-VTE3 co-expressed with At-VTE4	Transgenics	Van-Eenennaam et al. (2003)
2	Chickpea	β-carotene, leutin rich	1-4QTLs	QTL mapping	Abbo et al. (2005)
3	Groundnut	β-carotene, lutein and cryptoxanthin	Phytoene synthase 1 (*psy1*)	Transgenics	Bhatnagar et al. (2010)
		Anthocyanidin	a putative candidate gene and linked marker InDel02	eQTLmapping	Huang et al. (2006)
		Oleic acid	*ahFAD2* gene	MAS	Bera et al. (2018)
		Resveratrol	9 QTLs identified	ddRAD sequencing and High-Density genetic map	Luo et al. (2021)
4	Safflower (*Carthamus tinctorious*)	Gamma linolenic acid (GLA)	*Delta-6- desaturase* gene	Transgenics	Devi et al. (2008)
5	Mustard (*Brassica* spp.)	δ-tocopherol	*gamma-TMT* gene	Transgenics	Yusuf and Sarin, (2007)
		Carotenoid	*crtB*	Transgenics	Shewmaker et al. (1999)
		β-carotene, zeaxanthin, violaxanthin and lutein	Epsilon cyclase gene	RNAi technology	Yu et al. (2008)
6	Sunflower (*Helianthus annuus*)	Oleic acid	*FAD2*	Transgenics	Smith et al. (2007)
7	Canola	Protein-rich	*ACC7* gene	Transgenics	Roesler et al. (1997)

Omega-3 fatty acids are considered to be essential for brain development, which is mainly available through oil seed crops in human diet. The areas of crop improvement in terms of nutrition in oilseeds rely on improving oil quality, resveratrol content and improved shelf life (Pandey et al., 2014; Qi et al., 2014; Shasidhar et al., 2017; Luo et al., 2021). Profiling of nutraceutical properties of 60 groundnut cultivars differentiating in kernel colors has been carried out and marker-trait association studies have been carried out (Nayak et al., 2020). The expression of phytoene synthase showed 50-fold increased levels of carotenoids in rapeseed using genetic engineering (Shewmaker et al., 1999). An increased expression of zeaxanthin, violaxanthin and lutein by targeting the downregulation of the epsilon cyclase gene using RNAi technology) has been reported in mustard (Yu et al., 2008). The molecular mapping and QTL analysis of flavonoid genes was also elucidated in rapeseed (Qu et al., 2016), soybean (Li et al., 2016)**,** and groundnut (Mondal et al., 2015). Efforts are being carried out to use advanced biotechnological applications to improve oilseeds nutritionally for further crop improvement (Table 3).

### Fruits

Fruits are promoted as functional foods as they are a rich source of several antioxidants, polyphenols, minerals, soluble fibers, vitamins especially C, A and E. They primarily consist of flavonoids including flavonols, flavones, isoflavones, flavanones and anthocyanins, and non-flavonoid polyphenolics including phenolic acids, lignans and stilbenes (Joy et al., 2018). Though fruits are the major source of functional foods, systematic experimental reports on the utilization of omics technologies to improve functional components are limited to a few fruit crops (Table 4).

**TABLE 4:** Study of functional foods in fruits and vegetables using biotechnological approaches.

S. No	Crop	Functional food	Gene	Methodology	References
1	Orange	Lycopene	Carotenoid and MEP pathway genes	Mutation breeding	Alquezar et al. (2008)
2	Pummelo	Naringin	Naringin	*In vitro* and *In vivo* studies followed by molecular docking	Cheng et al. (2020)
3	Apple	Astaxanthin	*bkt* and *crt*R-B genes	Transgenics	Jia et al. (2019)
		Flavanols, anthocyanins and hydroxycinnammic acid	79 QTLs identified for 17 polyphenolic content	QTL mapping and candidate gene mapping	Chagné et al., 2012
4	Grapes	Flavonols, anthocyanin and tannins	*VviGST1, VviGST3, and VviGST4*	Transgenic	Pérez-Díaz et al. (2016)
5	Watermelon	Lycopene	2 candidate genes Cla005011 and Cla005012	MAS	Wang et al. (2019a)
6	Walnut	Walnut Protein Hydrolysate (WPH)	Walnut Protein Hydrolysate (WPH)	*Invitro* and *In vivo* studies	Wang et al. (2019b)
7	Strawberry	Total flavonoids	7 QTLs and 2 candidate genes (*Fa*MYB1 and *Fa*F3′H) controlling flavonoid content identified	QTL analysis and Expression studies	*Karmakar et al.* (2020)
8	Tomato	Anthocyanin	Anthocyanin 1 (*ANT1*)	TALENs and CRISPR/Cas9 achieved gene	Čermák et al. (2015)
		Anthocyanin	Phytoene desaturase (*SlPDS*),	CRISPR/Cas9	Pan et al. (2016)
		Lycopene	*Lycopene β/ε -Cyclase*	RNAi technology and Agrobacterium-mediated gene transformation	Ma et al. (2011)
		Carotenoid	*Brassicajuncea*3-Hydroxy-3-methylglutaryl-coenzyme asynthase (*Bj*HMGS)	Mutation breeding	Galpaz et al., 2008, Liao et al. (2018)
		Carotenoids, Vit-C, Vit-E and Phenolic acids	7 QTLs for carotenoids, 6 for Vit-C, 5 for Vit-E, 3 for Glutathione, and a total of 43 QTLs for phenolic acids were identified	QTL mapping	Colak et al. (2020)
9	Cabbage	Anthocyanin	Purple (*Pr*) gene (flavonoid 3′-hydroxylase, dihydroflavonol 4-reductase, and leucoanthocyanidindioxygenase)	Transgenics	Chiu et al. (2010)
10	Carrot	Carotenoid	*DCAR 032551* gene	Genome assembly and Transcriptomics	Iorizzo et al. (2016)
11	Bell pepper	Alkaloid compound- Capsaicinoids	Deaminase (TD) and prephenate aminotransferase enzyme identified	*de novo* transcriptome assembly	Liu et al., 2013, Bennett and Kirby, 1968
12	Potato	Essential amino acid-rich protein and rich in methionine	*AmA1*	Transgenics	Chakraborty et al. (2010)
		Inulin producing	Constitutive expression of the *1-SST* and *1-FFT* (genes of globe artichoke)	Transgenics	Hellwege et al. (2000)
		β-carotene and lutein rich	*crtB* gene	Transgenics	Ducreux et al. (2005)
13	Chilli	β-carotene	lycopene beta-cyclase (β-Lcy) gene	Transgenics	El Nagar, (2018)
14	Brinjal	β-carotene	*crt*B gene	Transgenics	Mishiba et al. (2020)
16	Sweet potato	α-tocopherol	tocopherol cyclase (IbTC)	Transgenics	Kim et al. (2019)
17	Broccoli	Sulforaphane	MAM1, myrosinase and FMO_GS–OX2_ genes	Transgenics	Cao et al. (2021)

In citrus, GWAS studies were conducted on 787 different citrus fruits using 1,841 SNP markers, and marker-trait associations were studied on fruit quality traits, including acid %, taste, and aroma (Minamikawa et al., 2017). Specific locus amplified fragment (SLAF) sequencing was performed over *C. reticulata* × *P. trifoliata* F_1_ pseudo testcross population and have constructed a high density integrated genetic map with 3,817 markers. This study has identified 17 significant QTLs of which three colocalized genomic regions were observed for multiple carotenoid constituents (Zheng et al., 2018). In another study, a navel orange (*Citrus sinensis* L. Osbeck) mutant (“Cara Cara”) was developed with bright red pulp with presence of lycopene (Alquezar et al., 2008). The expression analysis of genes involved in the carotenoid pathway using HPLC, northern hybridization, and RT-PCR indicated the increased accumulation of lycopene content in the mutant compared to navel orange. To elucidate the basis of lycopene accumulation in Cara Cara, the carotenoid profile and expression of three isoprenoids and nine carotenoid genes in flavedo and pulp of Cara Cara and Navel fruits throughout development and maturation were studied. The results indicated the accumulation of lycopene along with phytoene and phytofluene from early developmental stages in pulp as well as peel (Alquezar et al., 2008). Lemons are known for several functional components, including phenolics, vitamins, minerals, dietary fiber, essential oils and carotenoids (González-Molina et al., 2010). In the case of Sicilian blood oranges, retrotransposons were shown to induce seed-specific accumulation of anthocyanins during cold stress (Butelli et al., 2012). Edmunds et al. (2012) reported that the anti-inflammatory property of kiwifruit extract is due to the changes in the expression level of genes involved in the immune signaling pathway and metabolic processes using microarray technique.

The king of fruits “mango” (*Mangifera indica*) is a rich source of various polyphenolic compounds and is found in all the parts of the plant including pulp, peel, seed, bark, leaf, and flower. Mango polyphenols, especially mangiferin, acts as an antioxidant and has several health benefits (Masibo and He, 2008). The transcriptomics and proteomics studies in mango have predicted the involvement of genes involved in the anthocyanin biosynthesis pathway during the fruit development stage of mango (Wu et al., 2014). There is little effort towards the use of biotechnological approaches to improve the functional components of mango.

Red grapes are significant sources of anthocyanins, the main compounds responsible for the color of red grapes and wine (Mazza and Francis, 1995). Metabolite profiling of bioactive components of grapes especially flavonols, anthocyanins, and tannins indicated the presence of several bioactive compounds. The quercetin and kaempferol content was found to be greater in white grapes than red ones, but the red grapes were reservoirs of other bioactive components such as myricetin, laricitrin, syringetin and isorhamnetin (Mattivi et al., 2006). Resveratrol, an antioxidant that is known to lower blood pressure, and act as a chemopreventive with antiaging benefits are present in grapes. These flavonoids not only provide health benefits to humans but also help plants to fight against several biotic and abiotic stresses. For instance, transformation of bHLH transcription factor gene, *VvbHLH1* from grapes into *Arabidopsis,* resulted in an increased accumulation of flavonoids and enhanced salt and drought tolerance (Wang et al., 2016)*.*

In Japanese plum (*Prunus salicina*), the molecular marker associated with transcription factors found in the flavonoid pathway was used to study population diversity (González et al., 2016). Date palm (*Phoenix dactylifera*) fruits are composed of minerals (Se, Cu, K, and Mg), vitamins (C, A, B6, B9, B2, B3) besides being a good source of total phenolics and natural antioxidants (such as anthocyanins, ferulic acid). Phenolic compounds and selenium present in date fruit impart antioxidant activity (Guizani, 2013). Similarly, transcriptome sequencing in Indian gooseberry (*Phyllanthus emblica*) revealed the genes involved in flavonoid and vitamin C biosynthesis (Kumar et al., 2016). In many fruits, biotechnological approaches, including “omics” studies and use of molecular markers for trait mapping to improve bioactive components are very limited.

### Vegetables

Among vegetables, most of the genomics studies have been carried out in tomatoes as this crop is considered to be one of the model plants in genetic transformation and other genomics studies. The most critical functional component present in the tomato is carotenoids, especially lycopene and anthocyanins. To obtain lycopene-rich tomatoes, the genes encoding lycopene β/ε-cyclase, responsible for the conversion of lycopene to carotenoid, were silenced using RNAi technology. Significant increases in lycopene content were observed in transgenic plants (Ma et al., 2011). A mutation breeding approach was also used to increase the carotenoid content of tomatoes by 30%. Abscisic acid-deficient mutants in tomatoes have been shown to increase the lycopene content (Galpaz et al., 2008). Further, vegetables rich in anthocyanins were developed by overexpression of specific genes of the carotenoid biosynthesis pathway that induced a purple color, especially in tomato and cauliflower (*Brassica oleracea* var. botrytis) (Gonzali et al., 2009; Chiu et al., 2010).

Genome-editing technologies, especially CRISPR-Cas9, has potential use in horticultural crops (Karkute et al., 2017). Recently, this technique was used to edit five genes that are involved in the carotenoid pathway to increase lycopene content by inhibiting the conversion from lycopene to β- and α-carotene in tomatoes that increased lycopene content by five-folds (Li et al., 2018). In another study, intense purple-colored tomato plants were obtained by overexpressing an Anthocyanin mutant 1 (*ANT1*) gene that encodes for Myb transcription factors using TALENs and CRISPR/Cas9 approaches (Čermák et al., 2015). Furthermore, phytoene desaturase (*S1PDS*), an essential enzyme in carotenoid biosynthesis, and phytochrome interaction factor PIF 4 (*S1PIF4*) were targeted using gRNAs with the stable transformed CRISPR/Cas9 system (Pan et al., 2016). Most of the flavonoids in tomatoes are present in the peel of the fruit. Hence, a holistic approach of pathway engineering to increase the content of novel flavonoids especially stilbenes in the flesh of the tomato fruit was reported (Schijlen et al., 2006).

In carrot (*Daucus carota* subsp. Carota), a candidate gene, *DCAR_032551* that is responsible for carotenoid accumulation in carrot taproot and is co-expressed with several isoprenoid biosynthetic genes was identified from genome assembly and transcriptomic studies (Iorizzo et al., 2016). A candidate gene-based association study was carried out in carrots using 109 SNPs in 17 candidates/carotenoid biosynthesis genes over 380 diverse carrot cultivars, indicated the association of carotenoid content with the root color (Jourdan et al., 2015). A terpene synthase gene family of carrot was studied using QTL analysis and candidate gene-based association on a panel of carrot diversity set of 85 cultivars. GBS approach was used to genotype the panel with >168,000 SNPs (Keilwagen et al., 2017). Similarly, in bell pepper, several putative candidate genes are involved in the biosynthesis of capsaicinoids, such as Dihydroxyacid dehydratase (*DHAD*), Thr deaminase (*TD*) and Prephenate aminotransferase (*PAT*) were predicted from *de novo* transcriptome assembly (Liu et al., 2013).

Besides, several transcriptomics studies related to functional foods are available in crops such as lettuce (*Lactuca sativa*) (Zhang et al., 2017). In general, there is much scope to use genomics approaches to understand the molecular mechanisms and to increase the functional components in fruits and vegetables as evident by reports (Table 4).

### Spices and Condiments

In spices and condiments, several studies have been carried out to profile metabolites, especially flavonoids, tannins, and alkaloids (Lee and Shibamoto, 2001; Shahidi and Ambigaipalan, 2015). In cinnamon (*Cinnamomum verum*), DART-QToF-MS method was utilized to discriminate true cinnamon from other species (Avula et al., 2015). There are limited reports on trait mapping in the case of spices (Table 5).

**TABLE 5:** Study of functional foods in beverages, spices and condiments using biotechnological approaches.

S. No	Crop	Functional food	Gene	Methodology	References
1	Coffee	Caffeine	N-methyltransferase genes, CaMXMT1	RNA interference method, Transgenics	Ashihara et al. (2008)
			65 caffeine associated SNPs identified	Genome sequencing and KEGG pathway-based analysis	Tran et al. (2018)
2	Tea	epigallocatechingallate, epigallocatechin, epicatechingallate	*CsANR1* and *CsANR2*	Expression in *E. coli*	Pang et al. (2013) Kim et al. (2014)
		Catechins and polyphenols	Demethylase gene	Transgenics followed by metabolic engineering	Yadav et al. (2020)
		Caffeine	27 QTLS were mapped to 8 linkage groups	2b-RAD Sequencing and High-Density genetic mapping	Xu et al. (2018)
3	Cocoa	Catechins and proanthocyanidins	Glycerol-3-phosphate acyltransferase (*GPAT*) genes, and lysophospholipid acyltransferase (*LPAT*) genes	Expression studies in yeast	Wei et al. (2018)
4	Cardamom	d-limonene	d-limonene	RNA sequencing Transcriptomics	Nadiya et al. (2017)
5	Clove	Eugenol and eugenyl acetate	Metabolites extracts	Gas chromatography/mass spectrometry	Lee and Shibamoto et al. (2001)
6	Black Pepper	Piperine	Piperine	Transcriptomics	Hu et al. (2015)
7	Garlic	Organic sulfur compounds	Acetolactate synthase (*ALS*) gene	Transgenics using the biolistic method	Park et al. (2002) Santhosha et al. (2013) Al- Safadi et al. (2000)
8	Fenugreek	Saponins	*diosgenin*	Gene expression studies	Ciura et al. (2017)
9	Saffron	Crocin made up of Apo carotenoids	Carotenoids	Induced mutation (gamma rays and chemical mutation)	Khan et al., 2011; Kyriakoudi et al., 2015

Most of the research in spices is related to the discovery of functional components. For instance, garlic has organic sulfur compounds as primary functional foods that have medicinal properties to reduce common cold, blood pressure and harmful cholesterol levels (Martin-Lagos et al., 1995). The functional food in turmeric is referred as curcumin, which acts as a acid neutralizer, blood purifier, tonic and antiseptic. The biological properties of curcumin were explored by using protein expression studies (Fang et al., 2011). The functional component of cardamom (*Elettaria cardamomum*) is d-limonene with antibacterial, anti-inflammatory, analgesic, and antispasmodic activities (Nadiya et al., 2017). Similarly, eugenol and eugenyl acetate, the functional components of clove (*Syzygium aromaticum*) are natural oxidants (Lee and Shibamoto, 2001). Coumarin, a functional component of cinnamon at lower doses has blood-thinning, anti-fungicidal and anti-tumor activities (Kawatra and Rajagopalan, 2015). Piperine from black pepper (*Piper nigrum*) has antioxidant, anti-inflammatory, and anticancer properties (Gorgani et al., 2017). Cumin (*Cuminum cyminum*) has cuminaldehyde that enhances appetite, taste perception, digestion, vision, strength, and lactation. It is also used to treat diseases such as fever, loss of appetite, diarrhea, vomiting, abdominal distension, edema and puerperal disorders (Sowbhagya, 2013). Ginger (*Zingiber officinale*) has gingerols, shagols, and paradols with antioxidant, antimicrobial, and anti-inflammatory potential (Butt and Sultan, 2011). Nutmeg (*Myristica fragrans*) has tannin, flavonoid, and terpenoid which are natural antioxidants (Assa et al., 2014). Coriander (*Coriandrum sativum*) has carotenoids, polyphenols and essential oils, which provides vitamin A and vitamin C (Laribi et al., 2015). Fenugreek (*Trigonella foenum-graecum*) has quercetin, kaempferol and vitexin derivatives which are anti-diabetic and anti-nociceptive properties (Ghosh et al., 2015). Saffron (*Crocus sativus*) has crocins, picrocrocin, and safranal, which is antispasmodic, eupeptic, gingival sedative, carminative, diaphoretic activities (Melnyk et al., 2010). Using mutation breeding in saffron has increased yields (Khan et al., 2011) that in turn increases the overall bioactive components per plant.

Transgenic research is still an emerging area in spices and condiments. In garlic and turmeric (*Curcuma longa*), genetic engineering approaches were utilized for developing herbicide tolerant plants (Park et al., 2002; Shirgurkar et al., 2006). Although there are some reports on the transcriptome of black pepper fruits (Hu et al., 2015), ginseng (Panax ginseng) (Rai et al., 2016), and cardamom (Nadiya et al., 2017) to study global transcriptome, there are no reports related to functional components in most of the spices. There is tremendous potential to use genomics approaches including trait mapping, transcriptomics, whole-genome studies and allele mining in case of spices to demonstrate and increase the functional components.

### Beverages

Beverage crops produce potable beverages other than water. Major beverage crops include Coffee (*Coffea* spp.), Tea (*Camelia sinensis*), Cocoa (*Theobroma cacao*), and Lemongrass (*Cymbopogon citratus*). Coffee has caffeine as the primary phenolic compound and is known to reduce the risk of stroke and cancer. Caffeine in higher doses is harmful as it may lead to insomnia, nervousness, restlessness, irritability, an upset stomach, a fast heartbeat, and even muscle tremors. As a result, there are efforts to improve decaffeinated coffee plants using RNAi technology (Ashihara et al., 2008). Tea has catechins and epicatechin as primary functional foods, and they are known to possess chemopreventive activities against prostate and ovarian cancers, anti-obesity and anti-diabetic effects. Efforts are underway to elucidate the proanthocyanidin pathway, also to reduce caffeine content (Pang et al., 2013). Lemongrass has citral as its primary functional food which has antimicrobial and medicinal properties. Little research has been performed on this crop.

QTLs for flavonoid-related traits in a tea were identified using a high-density genetic map (Xu et al., 2018). Several transcriptomics studies have been carried out in tea to elucidate genes involved in polyphenol synthesis, Catechin biosynthesis and other regulatory networks (Mamati et al., 2006; Wu et al., 2016; Sun P. et al., 2017). To knock down the expression of the genes involved in caffeine biosynthesis, RNAi was used to repress the expression of the gene encoding theobromine synthase (*CaMXMT1*) that reduced the caffeine content in the transgenic coffee plants up to 70% (Ogita et al., 2003). In the case of tea, the functional characterization of the proanthocyanidin pathway and potential applications in metabolic engineering was elucidated (Pang et al., 2013). Cocoa rich in catechins and proanthocyanidins has a promising effect on lowering blood pressure, boosting moods, and sharpening memory. Metabolic engineering of yeast for cocoa butter production was attempted by cloning the genes involved in triglycerol synthesis viz., glycerol-3-phosphate acyltransferase (GPAT), lysophospholipid acyltransferase (LPAT) from cocoa into yeast (Wei et al., 2018). Efforts are being made to develop lemongrass varieties such as Jor Lab L-8 with higher amounts of essential oil and herbage production (Mohan et al., 2016). The biotechnological applications have not been effectively utilized to increase the functional components in beverages and there are few reports related to this (Table 5).

### Medicinal Plants

Medicinal plants are called so because of their antibiotic, antidiabetic, antihyperglycemic, and antihyperlipidemic properties. Most medicinal plants are not consumed as staple foods, but as preventive medicines for several diseases ranging from the common cold to complex diseases like cancer. Herbal genomics has high potential to explore, though there are few efforts related to molecular breeding and genetic engineering in the medicinal crops (Chakraborty, 2018). However, metabolite profiling of some medicinal plants has been studied. In the case of a famous Ayurvedic crop Haritaki (*Terminalia chebula*), a component of Triphala (an ayurvedic composition), the metabolite profiling of polyphenols and evaluation of the decoction as a chemopreventive agent was studied (Pellati et al., 2013). Similarly, metabolite profiling was examined in a highly traded South African medicinal plant commonly known as pain brush lily (*Scadoxus puniceus*) and the bioactive compounds were isolated (Naidoo et al., 2018). Efforts for profiling polyphenols, alkaloids and other bioactive compounds are being carried out in other Asian medicinal plants (Gibon et al., 2006; Vega-Gálvez et al., 2011; Gantait et al., 2014; Hao and Xiao, 2015; Saito, 2018). For instance, in the case of Candyleaf (*Stevia rebaudiana*), the water extracts from leaf and calli were shown to have antioxidant activity and contain bioactive compounds including folic acid, vitamin C, catechin, quercetin and pyrogallol. Higher reactive oxygen species (ROS) scavenging activities were found in leaf extracts (Kim et al., 2011). Transcriptomics studies have also been carried out in some of the important medicinal plants including Ashwagandha (*Withania somnifera*) to understand the secondary metabolites which have therapeutic utilization (Tripathi et al., 2020).

Recent advances in metabolite and pathway engineering and their utilization in medicinal plant research have positively contributed to herbal genomics research. Most of the molecular studies in medicinal plants involved either discovery of the genes/enzymes/pathways related to secondary metabolites or increasing the production of the secondary metabolites using elicitors, hairy root cultures or metabolite engineering approaches.

## Future Prospects

Current approaches in crop sciences using integrated omics platform aims at providing a nutritionally rich, diverse balanced diet to the society. Several leading edge technologies in understanding and manipulating different segments of scientific research areas *viz.* genomics, proteomics, metabolomics *etc.* has enabled the researchers to enhance contents of key nutrients in crop plants. Not just nutrition, but reducing the unflavorful compounds (phytic acid, acrylamide-forming amino acids, *etc.*) in food crops has allowed people to consume a wide range of food crops. Bio fortification has potential to solve nutrition deficiencies and in this view several food crops *viz.* rice, maize, wheat, *etc.* have been biofortified to have enhanced amounts of Fe, Zn, *etc.* (Ye et al., 2000; Gil-Humanes et al., 2014; Mugode et al., 2014; Trijatmiko et al., 2016). Crop improvement with new advancements in field phenomics, employing applications of machine learning (Niazian and Niedbala., 2020), nanotechnology and artificial intelligence (Ben Ayed and Hanana, 2021; Zhang et al., 2021), biosensors like lidar (Jin et al., 2021) followed by statistical analysis using data science (Tong and Nikoloski, 2021) approaches will enable researchers to precisely assess traits for plant breeding and development (Deery and Jones, 2021).

In order to ensure the nutrional security, along with enhancing the nutritional value, we need to work on reduced food-wastes that has a significant economic, environmental and social impact (FAO, 2019). Several initiatives in estimating food waste and prevention has been proposed (Moraes et al., 2021), however, devising methodologies in estimating and reducing food wastage is still a paradox (Richards et al., 2021). This can be featured as an opportunity to overcome malnutrition in addition to food waste reduction and stabilize bio-economy with sustainable processing of food waste into bio-based products (Sharma et al., 2021). The innovative technologies for extraction and microencapsulation of bioactives using novel technologies in metabolomics can be utilized in enhancing plant based functional foods (Pattnaik et al., 2021).

The research in nutrition and omics technologies in food science with epidemiological techniques should be classically established (Palou et al., 2004). In the future, the advances in foodomics and nutrigenomics can enable to achieve nutritional security in most of the crops. Utilization of omics technologies to identify the functional components in less explored crops like fruits, vegetables, spices and medicinal plants is essential to improve the functional components. There is a need to integrate multi-omics technologies in functional food research to elucidate and enhance the nutrition components in plants. Nutrigenomics can provide insights into the interaction of functional foods in human health and would provide allusion towards scientifically personalized diet.

## Data Availability

The raw data supporting the conclusions of this article will be made available by the authors, without undue reservation.
